# Expression of Sumoylation Deficient Nkx2.5 Mutant in Nkx2.5 Haploinsufficient Mice Leads to Congenital Heart Defects

**DOI:** 10.1371/journal.pone.0020803

**Published:** 2011-06-03

**Authors:** Eun Young Kim, Li Chen, Yanlin Ma, Wei Yu, Jiang Chang, Ivan P. Moskowitz, Jun Wang

**Affiliations:** 1 Program in Genes and Development, University of Texas Health Science Center at Houston, Houston, Texas, United States of America; 2 Department of Basic Research Laboratories, Texas Heart Institute, Houston, Texas, United States of America; 3 Institute of Biosciences and Technology, Texas A&M Health Science Center, Houston, Texas, United States of America; 4 Department of Biochemistry and Molecular Biology, University of Houston, Houston, Texas, United States of America; 5 Departments of Pediatrics and Pathology, The University of Chicago, Chicago, Illinois, United States of America; Brigham and Women's Hospital, United States of America

## Abstract

Nkx2.5 is a cardiac specific homeobox gene critical for normal heart development. We previously identified Nkx2.5 as a target of sumoylation, a posttranslational modification implicated in a variety of cellular activities. Sumoylation enhanced Nkx2.5 activity via covalent attachment to the lysine residue 51, the primary SUMO acceptor site. However, how sumoylation regulates the activity of Nkx2.5 in vivo remains unknown. We generated transgenic mice overexpressing sumoylation deficient mutant K51R (conversion of lysine 51 to arginine) specifically in mouse hearts under the control of cardiac α-myosin heavy chain (α-MHC) promoter (K51R-Tg). Expression of the Nkx2.5 mutant transgene in the wild type murine hearts did not result in any overt cardiac phenotype. However, in the presence of Nkx2.5 haploinsufficiency, cardiomyocyte-specific expression of the Nkx2.5 K51R mutant led to congenital heart diseases (CHDs), accompanied with decreased cardiomyocyte proliferation. Also, a number of human CHDs-associated Nkx2.5 mutants exhibited aberrant sumoylation. Our work demonstrates that altered sumoylation status may underlie the development of human CHDs associated with Nkx2.5 mutants.

## Introduction

Nkx2.5, a cardiac specific homeobox gene, is one of the earliest markers for cardiac progenitor cells and is central for normal fetal cardiac structural morphogenesis. Nkx2.5 null mice died from cardiac malformations [1], [2], while mice with Nkx2.5 haploinsufficiency caused atrial septal defects (ASDs), one of the most common forms of congenital heart diseases (CHDs) in humans, although the penetrance was low and was influenced by the mouse strain [3], [4]. The critical role of Nkx2.5 in the conduction system was revealed by observations in both human patients with heterozygous Nkx2.5 mutants and in animal models [5], [6]. Overexpressing wild type Nkx2.5 or one of its homeodomain (HD) mutants such as I183P in murine cardiomyocytes under the control of β-myosin heavy chain promoter resulted in embryonic lethality and/or heart failure, which was a consequence of dilated cardiomyopathy and/or a defective conduction system [7]. Transgene expression of the I183P mutant, driven by α-myosin heavy chain promoter (α-MHC), also led to cardiac dysfunction, but with later onset [8] and with phenotypic discrepancy [8], [9]. Postnatal cardiac specific ablation of Nkx2.5 by tamoxifen-inducible Cre expression starting at 2 weeks of age led to a conduction defect and dilated cardiomyopathy [10]. In the clinic, a multitude of heterozygous Nkx2.5 mutants were shown to be etiologically implicated in nonsyndromic CHDs [11]. Thus, Nkx2.5 has an essential role in the formation of cardiac structure and in the maintenance of cardiac function.

Nkx2.5 is a moderate activator of its target genes. In addition to its physiological activity achieved via DNA binding through its HD, it functioned through associations with other transfactors such as SRF, GATA4 and TBX proteins [12], [13], [14], [15], [16], [17]. It is also noteworthy that the transcriptional activity of Nkx2.5 was modulated in part by posttranslational modification such as phosphorylation by casein kinase II (CKII) and sumoylation [18], [19]. Sumoylation is a posttranslational modification in which the SUMO moiety is covalently and reversibly conjugated to the lysine residue(s) located in the target protein. Sumoylation on lysine 51 of the Nkx2.5 protein substantially increased its activity, and mutation of lysine 51 to arginine (K51R) suppressed SUMO linkage and the activity of Nkx2.5 [18]. However, how the sumoylation deficient Nkx2.5 mutant behaves in vivo is unknown.

Recent studies pointed to an important role of SUMO conjugation in cardiac structural morphogenesis and dysfunction [20], [21]. SUMO-1 knockout (KO) mice exhibited cardiac structural defects, although the phenotypic penetrance may have been affected by genetic background [22]. Nkx2.5 played a role in the development of SUMO-1 KO-induced CHDs, because the compound Nkx2.5^+/−^/SUMO-1^+/−^ mice exhibited more severe cardiac phenotypes than the mice with single heterozygous knockout of either Nkx2.5 or SUMO-1 [22]. Given the above findings and the effect of sumoylation on transcriptional activity of Nkx2.5 [18], we asked whether CHD-linked Nkx2.5 mutants exhibited abnormal sumoylation activity and how defective sumoylation of Nkx2.5 affected cardiogenesis in vivo. We uncovered that sumoylation was decreased in a number of naturally occurring Nkx2.5 mutants, including those HD mutants tested, and that cardiac restricted expression of the sumoylation deficient mutant, K51R, in the Nkx2.5 haploinsufficient (Nkx2.5^+/−^) mice led to CHDs.

## Materials and Methods

### Ethics Statement

The animals were handled in accordance with institutional guidelines with appropriate approvals (protocol ID 09018) by IACUC at Texas A&M Health Science Center.

### Generation of transgenic and knockout mice

The human influenza hemagglutinin (HA) tagged K51R mutant was generated as reported [18] and then was subcloned between the 5.4 kb mouse α-MHC promoter (provided by Dr. J. Robbins, University of Cincinnati) and the Simian virus 40 (SV40) polyadenylation sequence via SalI sites. The orientation of the inserted transgene was confirmed by DNA sequencing. The construct was microinjected into the pronucleus of fertilized eggs from FVB mice, and offspring were screened by polymerase chain reaction (PCR). One mouse line was identified to transmit detectable transgene. F0 founder was crossed back to C57BL/6 and scored for positive transmission revealed by PCR using the following oligos (5′ to 3′): forward, CGGCACTCTTAGCAAACCTC; reverse, CACCGCCTTCTGCAGCGC. The expression level of the transgene was evaluated by western blot. K51R-Tg offspring were then crossed back to C57BL/6 for one to four generations. Thus, the animals obtained from this cross were examined under the mixed genetic background of FVB and C57BL/6. The generation of Nkx2.5 knockout (Nkx2.5^+/−^) mouse line was detailed previously [23], in which one Nkx2.5 allele was disrupted by the homologous recombination of Cre recombinase cDNA after translation start codon ATG. The cross breeding between K51R-Tg and Nkx2.5^+/−^ mouse lines was performed as needed. Genomic DNA was isolated from tail biopsies performed on weaned animals (about 3-week old pups) and screened by PCR. The animals were handled in compliance with institutional guidelines at Texas A&M Health Science Center at Houston Campus.

### Antibodies

Antibodies against Nkx2.5, HA and GAPDH were purchased from Santa Cruz Biotechnology, Inc., and anti-flag antibody (M2) was from Sigma.

### Plasmid construction, cell culture, transient transfection and western blot

The cardiac α-actin promoter driven luciferase reporter construct (Ca-actin-Luc), the expression vector encoding HA-tagged wild type Nkx2.5 and flag-tagged SUMO-1 was detailed previously [15], [24]. His_6_-tagged SUMO-1 cDNA was a gift from Dr. Hay RT (University of Dundee, UK) and was subcloned into PCGN vector on XbaI and KpnI sites. All Nkx2.5 point mutations, R25C (conversion of arginine 25 to cysteine), T177M (conversion of threonine 177 to methionine), N187K (conversion of asparagine 187 to lysine), R188G (conversion of arginine 188 to glycine), Y190C (conversion of tyrosine 190 to cysteine), C265Y (conversion of cysteine 265 to tyrosine), V309L (conversion of cysteine 309 to leucine), were generated using Stratagene mutagenesis kit based on manufacturer's protocol. Mutations were confirmed by sequencing. For cell culture, Hela cells were maintained in Dulbecco's Modified Eagle's Medium containing 10% fetal bovine serum. Transfections were performed on Hela cells cultured on either 6 cm plates for western blot assay or 12 well plates for luciferase activity assay using Lipofectamine 2000 based on the protocol provided by the supplier. For western blot analysis, 100 µg of protein extracts obtained from mouse hearts was subjected to NuPage SDS gel (Invitrogen) and transferred to PVDF membrane, which was labeled with desired antibodies and revealed by chemiluminescence. Luciferase activity assays were performed as previously described [18], [25]. Briefly, 200 ng of Ca-actin-Luc was co-transfected with 1 µg of expression vector encoding wild type Nkx2.5, or one of its mutants R25C, T177M, N187K, R188G, Y190C, C265Y, V309L, in the absence or presence of two dosages of flag-SUMO-1 (0.5 µg and 0.75 µg, respectively). The empty vector was used to balance the total amount of plasmids used in the reporter assay. Luciferase activity was assessed after 48 h transfection using Monolight™ 3010 (Pharmingen). Promoter activity was expressed as the ratio of luciferase activity induced by the presence of specific factor(s) to the control group with the presence of only empty vector. Data shown were expressed as mean ± SEM from at least two independent assays, with each carried out in duplicate.

### In vivo sumoylation assay and Ni^2+^-NTA chromatography

In vivo sumoylation assay and Ni^2+^-NTA chromatography were detailed previously [18], [25]. For in vivo sumoylation assay, the expression vectors for wild type Nkx2.5 or Nkx2.5 mutants were transfected into Hela cells cultured on 6 cm plates in the presence or absence of encoding vector for flag-tagged SUMO-1. Whole cell lysates were purified 48 hours after transfection in the presence of 25 mM isopeptidase inhibitor N-ethylmaleimide (NEM), which prevents desumoylation of those SUMO-conjugated substrates. 40 µg of prepared proteins from each group were subsequently analyzed in NuPage SDS gel, transferred to PVDF membrane, which was probed with desired antibodies and revealed by chemiluminescence. For Ni^2+^-NTA chromatography, Hela cell lysates containing expressed Nkx2.5 wild type or one its mutants in the presence of His_6_-tagged SUMO-1 were precipitated by Ni^2+^-NTA beads, and subsequently subjected to NuPage SDS gel. The blot was probed with anti-Nkx2.5 antibody and revealed by chemiluminescence. The relative level of sumoylated Nkx2.5 wt or its mutants was determined by quantification followed by normalization of the level of SUMO-conjugated Nkx2.5 to the level of free Nkx2.5 protein. The level of sumoylated wt Nkx2.5 was taken as 100%. The data from two independent assays were analyzed using software ImageJ (National Institutes of Health).

### Histopathology

Mouse neonatal hearts were dissected and fixed in 4% paraformaldehyde (PFA) at 4°C for overnight. Hearts were sectioned transversely at 10 µm thickness, which were stained with Hematoxylin and Eosin (H&E) according to the standard protocol.

### Immunohistochemistry

The mouse hearts at P7 were fixed in 10% formalin prepared in 1× phosphate-buffered saline (PBS), dehydrated and embedded in paraffin for histological sections. For antigen retrieval, 5 µM sections were boiled in sodium citrate buffer (10 mM, PH6.0) for 10 minutes, and then blocked in 2% bovine serum albumin (Vector Laboratories, SP-5050)/PBST (1XPBS/0.05% Tween20), followed by incubation with the primary antibodies monoclonal anti-cardiac troponin T (cTnT, 1∶200, Lab Vision, MS-295-P) and rabbit anti-Ki67 (1∶200, Abcam, ab15580) in blocking solution at 4°C overnight. Sections were then washed, followed by incubation with 1∶500 Alexa fluor® 594 goat anti-rabbit secondary antibody (Invitrogen, A31632) and Alexa fluor® 488 donkey anti-mouse secondary antibody (Invitrogen, A21202) in blocking solution at RT for 30 minutes. Sections were mounted with Vectashield® with DAPI (Vector Laboratory) and photographed under a Leica fluorescent microscope. TUNEL staining was conducted according to manufacturer's protocol (Promega). The number of double Ki67 positive or TUNEL positive and cTnT positive cells (Ki67+/cTnT+ or TUNEL/cTnT+) was scored in at least three randomly selected fields in the left ventricle or atrioventricular septum and expressed as mean ± SEM.

### Echocardiography

Two dimensional M-mode of Vevo 770 (Visual Sonics, Toronto, Canada) was used to measure the cardiac function on mice of interest with various ages as designed. Briefly, mice were anesthetized by inhalation of 1% isofluorane and fixed to a warm pad. Chest hair was removed and contacted with the probe to record cardiac function indices. Evaluations of cardiac function and heart dimensions were performed by an expert analyst blinded to the genotype of animals.

### Reverse transcription-quantitative PCR (RT-qPCR)

RNAs were extracted from K51R-Tg and age-matched control mouse hearts using Trizol based on manufacturer's protocol. 1 or 5 µg total RNAs were used for reverse transcription reaction using cloned reverse transcriptase (Invitrogen), followed by quantitative PCR on machine MX3000 (Strategene) using specifically designed probes for the particular genes of our interest. The sequences (5′ to 3′) of oligos used in this study were shown below: ANF: forward, gtgtccaacacagatctgatggat; reverse, gcctagtccactctgggctccaat; BNF: forward, gaaggtgctgcccagatgattct; reverse, gacggatccgatccggtctatctt; cardiac α-actin [26] : forward, cctctttcattggtatggaatct; reverse, gtacaatgactgatgagagatgg; cardiac α-MHC [27]: forward, ggaagagtgagcggcgcatcaagga; reverse, tctgctggagaggttattcctcgt; cardiac β-MHC: forward, ggccaacaccaacctgtccaagtt; reverse, tgcaaaggctccaggtctgagggc; MLC-2v [28]: forward, gccaagaagcggatagaagg; reverse, ctgtggttcagggctcagtc; skeletal α-actin [29] : forward, agacaccatgtgcgacgaaga; reverse, ccgtccccagaatccaacacga; SERCA forward, gagtcgaccagtcaattcttac; reverse, tcagtattaactccagttgcca; connexin 40: forward, atctcccacattcgttattg; reverse, aggaagatcccatagaggag; conexin 43 [30]: forward, taccacgccaccaccggccca; reverse, cattttggctgtcgtcagggaa. GAPDH: forward, atgttccagtatgactccactcac; reverse, gaagacaccagtagactccacga.

### Statistical analysis

The unpaired Student's *t* test, Chi-square test was applied to determine statistical significance between groups when applicable and shown in each Figure legend.

## Results

### CHDs-linked Nkx2.5 mutants displayed aberrant SUMO modification

**Figure 1:**
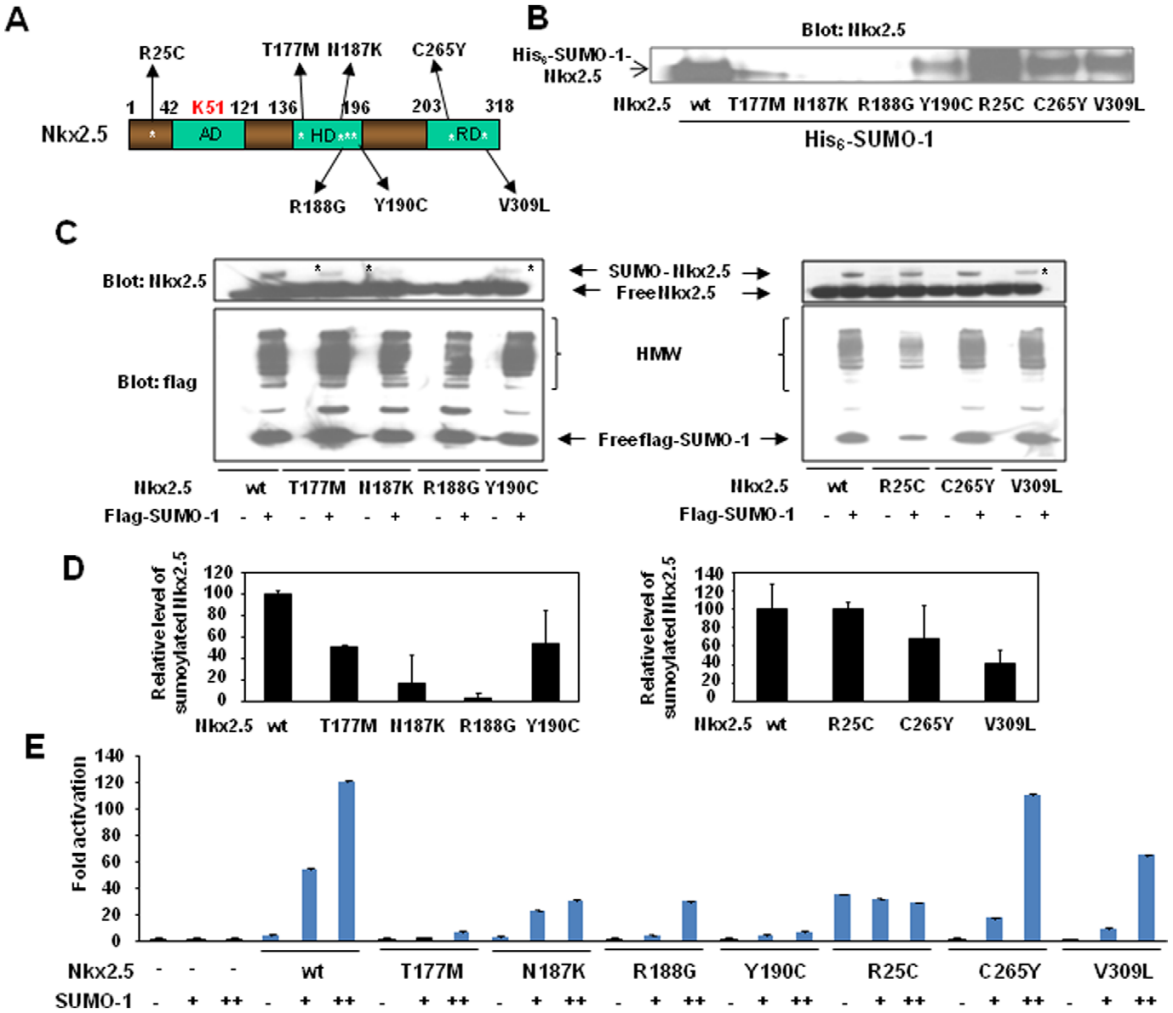
CHDs-associated Nkx2.5 mutants displayed aberrant SUMO modification. A. Schematic illustration of Nkx2.5 wild type and seven point mutants selected for sumoylation assays. Asterisks * indicated the relative locations of mutations. These seven point mutants generated on mouse Nkx2.5, R25C, T177M, N178K, R188G, Y190C, C265Y, and V309L, were equivalent to the following corresponding human missense Nkx2.5 mutants, respectively: R25C, Arg-Cys; T178M, Thr-met; N188K, Asn-Lys; R189G, Arg-Gly; Y191C, Tyr-Cys; C270Y, Cys-Tyr; V315L, Cys-Leu. See text for details. AD, activation domain; HD, homeodomain; RD, repression domain. B. Aberrant sumoylation was observed in Nkx2.5 mutants. Ni^2+^-NTA chromatography was performed on Hela cell lysates expressing wt Nkx2.5 or one of those mutants in the presence of His_6_-tagged SUMO-1. The representative data from three independent assays were shown. The blot was probed with anti-Nkx2.5 antibody. Arrow indicated His_6_-SUMO-1-conjugated Nkx2.5. C. Decreased sumoylation (indicated by *) was observed on all of those four HD mutants and V309L. Western blots were performed on cell lysates purified from Hela cells transfected with encoding vectors as indicated. Upper blot: anti-Nkx2.5; lower blot: anti-flag. In the upper panel, upper and lower arrows indicated free and flag-SUMO-1-conjugated Nkx2.5, respectively; in the lower panel, arrows indicated free flag-tagged SUMO-1. HMW, high molecular weight conjugates. The representative data from two independent assays were shown. D. Summary of the effects of Nkx2.5 missense mutations on sumoylation activity. Relative level of sumoylated Nkx2.5 wt and its mutants was obtained by normalization of the level of sumoylated Nkx2.5 to the level of respective free Nkx2.5 protein. The level of sumoylated wt Nkx2.5 was considered as 100%. The data from two independent assays were analyzed using software ImageJ (National Institutes of Health). E. SUMO-enhanced activity was impaired in those Nkx2.5 mutants associated with suppressed sumoylation. Luciferase reporter assays were conducted as described in the section of Materials and Methods. Note that R25C exhibited highest activity in the absence of exogenous SUMO-1, and that the addition of external SUMO-1 did not significantly increase its activity. Promoter activity was expressed as the ratio of luciferase activity induced by the presence of specific factor(s) to the control group with the presence of only empty vector. Data shown were expressed as mean ± SEM from at least two independent assays, with each carried out in duplicate. Nkx2.5, 1 µg. +, 0.5 µg, ++, 0.75 µg.

As an initial step to investigate whether altered sumoylation status underpinned those Nkx2.5 mutants that were associated with CHDs, sumoylation assay using Ni^2+^-NTA chromatography was performed on Hela cell lysates expressing His_6_-tagged SUMO-1 and Nkx2.5 wild type (wt) or one of seven point mutants created on murine Nkx2.5 protein, R25C, T177M, N187K, R188G, Y190C, C265Y and V309L (Fig. 1A). These point mutants corresponded to the following seven missense human Nkx2.5 mutants respectively that were previously described and were involved in human CHDs [5], [31]: R25C, T178M, N188K, R189G, Y191C, C270Y and V315L. As shown in Fig. 1B, four Nkx2.5 HD mutants tested, T177M, N187K, R188G, Y190C, showed significant decrease in sumoylation, while R25C still exhibited robust sumoylation. To better evaluate the changes in sumoylation activity of Nkx2.5 mutants vs. wt, western blots, in which loading controls were better monitored, were carried out on cell lysates purified from transfected Hela cells expressing Nkx2.5 wt or one of its mutants in the absence or presence of flag-epitoped SUMO-1, as indicated in Fig. 1C. Also, the relative level of sumoylated Nkx2.5 wt or mutants after normalization to the level of corresponding free Nkx2.5 protein was objectively determined using software ImageJ (Fig. 1D). While Nkx.2.5 wt and those mutants tested were expressed at comparable levels, N187K and R188G abolished over 80% sumoylation compared with Nkx2.5 wt, and T177M, Y190C and V309L blocked ∼50% sumoylation (Fig. 1C&D). Also, C265Y showed ∼30% decrease in SUMO linkage. Among these mutants, R25C appeared to have a normal sumoylation activity, however, this “normal” sumoylation status of R25C was achieved with much lower expression level of SUMO-1 (Fig. 1C. right panel), indicative of R25C's high sumoylation affinity.

Since SUMO conjugation promoted the capacity of Nkx2.5 to activate target gene promoters [18], we next compared the transcriptional activities of Nkx2.5 wt and those mutants in the presence of SUMO-1. Luciferase activity assays were performed on Hela cell lysates expressing Ca-actin-Luc along with Nkx2.5 wt or one of those above-mentioned mutants in the absence or presence of increasing dosages of SUMO-1 expression vector as indicated (Fig. 1E). In line with the observation from sumoylation assays (Fig. 1C&D), SUMO-1 substantially increased the capacity of Nkx2.5 wt to activate target gene promoter up to ∼120 fold, but this increase was impaired by the HD mutations. C309L mutant also failed to fully respond to SUMO-1. SUMO-1 enhanced the capability of C265Y mutant to the equivalent level as Nkx2.5 wt to stimulate reporter activity. Intriguingly, R25C alone exhibited higher ability to activate the target promoter by ∼34 fold, compared with ∼4 fold activation by wt Nkx2.5, and the addition of SUMO-1 did not further enhance the transcriptional activity of R25C. Taken together, these observations indicated that defective SUMO modification underlied the altered activity of four HD mutants and one C-terminal mutant (V309L) examined.

### Transgene expression of sumoylation deficient Nkx2.5 K51R mutant in the murine heart

**Figure 2:**
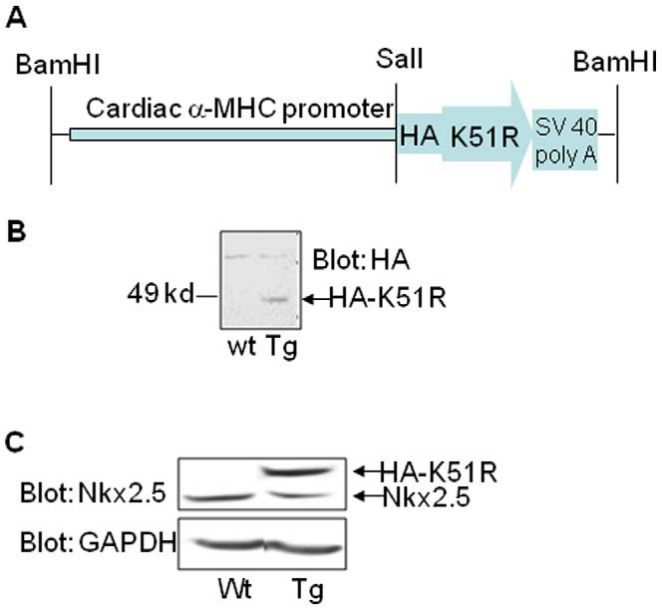
Generation of K51R transgenic (K51R-Tg) mice. A. Structural illustration of construct for generating K51R-Tg mouse line. B. Transgene HA-tagged K51R was detected in the transgenic heart. Western blot was carried out on heart lysates extracted from both wild type and K51R-Tg mice. The blot was probed with anti-HA antibody and revealed by chemiluminescence. Note that the HA-epitoped K51R was detected in Tg heart sample (arrow) but not in wild type heart sample. C. Evaluation of the expression level of transgene HA-tagged K51R. Western blot was performed as in B and the blot was reacted with anti-Nkx2.5 antibody first (Upper panel), then stripped and re-probed with anti-GAPDH antibody as a control (lower panel).

Since a number of CHDs-associated Nkx2.5 mutants showed defective sumoylation, we asked whether sumoylation deficient Nkx2.5 mutant K51R [18] could be causally linked to CHDs once expressed in vivo. We generated cardiomyocyte specific expression of HA-tagged K51R transgene (K51R-Tg) under the control of cardiac α-MHC promoter (Fig. 2A). One founder of K51R-Tg mouse transmitted detectable HA-epitoped K51R transgene in murine hearts (Fig. 2B). The expression level of HA-tagged Nkx2.5 was approximately 1.5∼2 folds compared with that of Nkx2.5 protein in the wild type mouse heart, as determined by protein blot using a specific antibody against Nkx2.5 (Fig. 2C). The offspring from the cross between K51R-Tg and wild type C57BL/6 mice were born at the expected Mendelian ratio (∼50%), indicating no embryonic loss. Except that two unusual premature deaths occurred at postnatal day (P) 2 and 9 (P2 and P9), which carried atrial septal defect (ASD) (data not shown), all other K51R-Tg mice appeared normal and fertile.

**Figure 3:**
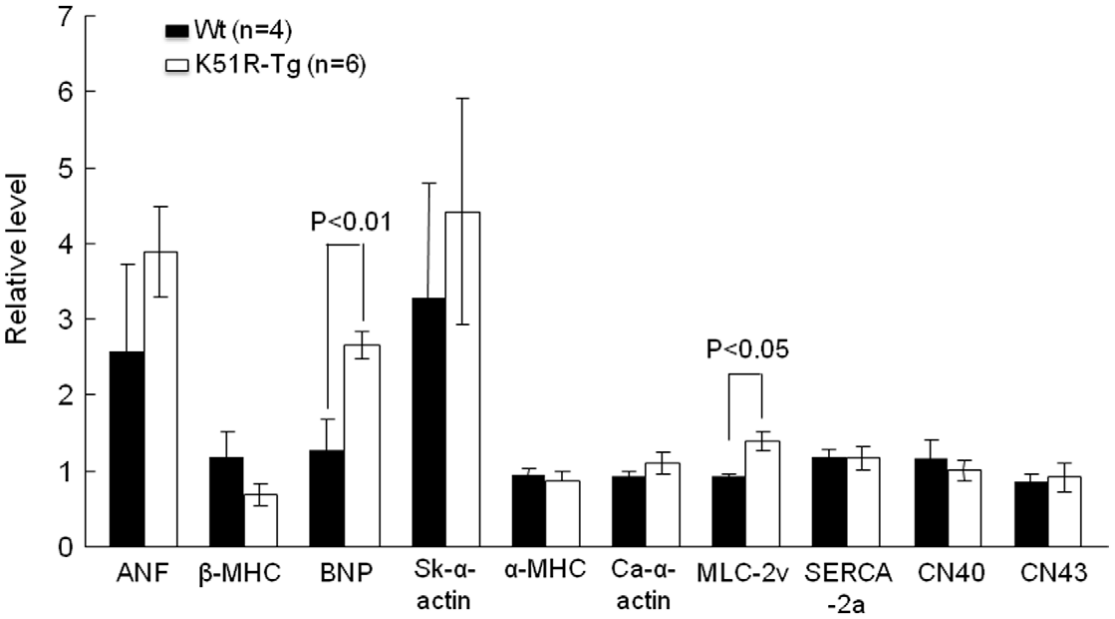
Dysregulation of cardiac genes in K51R-Tg mice. RT-qPCR revealed altered transcription of some cardiac genes examined such as BNP and myosin light chain (MLC)-2v in K51R-Tg hearts (n = 6) compared with those of control hearts (n = 4) at P50. RT-qPCR was performed as described in Materials and Methods. Unpaired student's *t* test was used for statistically significant analysis.

To gain an insight into cardiac functions in the mice bearing the K51R mutant, echocardiography was performed on K51R-Tg and wild type mice at ages of P50 and P300, respectively, using Vevo 770. Surprisingly, overexpression of the sumoylation resistant Nkx2.5 mutant in mice did not lead to overt defective cardiac functions (data not shown). In addition, the cardiac index LV mass/body weight ratio also showed no significant difference between the two age-matched groups (data not shown). Despite no discernable cardiac phenotype(s) in the K51R-Tg mice as evaluated by cardiac function, transcripts of cardiac genes such as cardiac brain natriuretic peptide (BNP) and myosin light chain 2v (MLC-2v) were elevated in the K51R-Tg hearts at P50 compared with those in the age-matched control hearts (Fig. 3). However, no significant changes were observed for the transcription of conduction function-involving genes such as connexin 40 and connexin 43, which were dysregulated in other mice carrying overexpressed Nkx2.5 proteins [7], [8]. Collectively, overexpression of K51R mutant in murine hearts caused dysregulation of a subset of cardiac gene transcriptions, but was insufficient to induce evident dysfunctional cardiac phenotypes, at least before age of one year.

### Compound K51R-Tg/Nkx2.5^+/−^ mice exhibited CHDs

**Figure 4:**
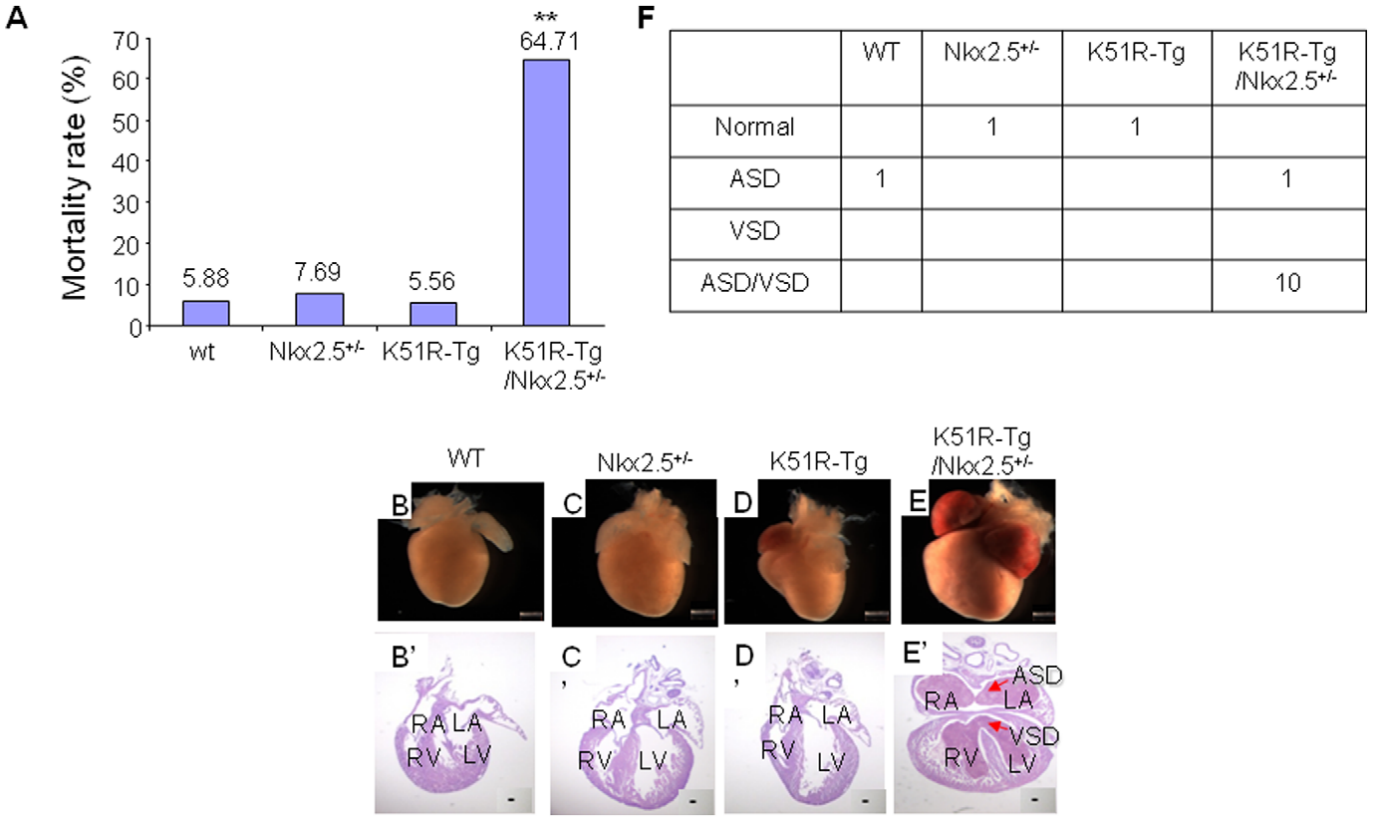
Compound K51R-Tg/Nkx2.5^+/−^ mice exhibited ASD and/or VSD. A. Mortality rate of compound K51R-Tg/Nkx2.5^+/−^ mice was significantly higher than those of other genotype mice. The data were collected from 65 animals of 7 litters. The number shown above each bar indicated the mortality rate of that corresponding group. B–E, Representative gross view of heart morphologies in littermate wt, Nkx2.5^+/−^, K51R-Tg and compound K51R-Tg/Nkx2.5^+/−^ mice at P4 was shown. Note that the atria of the compound K51R-Tg/Nkx2.5^+/−^ mouse heart were filled with blood. A′–D′, H&E staining on 10 µm sections of hearts corresponding to A–D was shown. Arrows in D′ indicated ASD and VSD, respectively. Scale bar, 100 µm. F. Incidence of cardiac defects in the demised mice from four genotype groups. Chi square test was used for statistical analysis. **, p<0.01, vs. other groups. RA, right atrium; LA, left atrium; RV, right ventricle; LV, left ventricle; ASD, atrial septal defect; VSD, ventricular septal defect.

In clinic, all Nkx2.5 allele mutations identified in human so far were heterozygous [32]. We asked whether K51R could induce similar CHDs in the presence of Nkx2.5 haploinsufficiency. The compound K51R-Tg/Nkx2.5^+/−^ mice were obtained after cross-breeding between K51R-Tg and Nkx2.5^+/−^ mice. The compound K51R-Tg/Nkx2.5^+/−^ mice were born in an expected Mendelian ratio (26.15%), however, the mortality rate in the first 14 days after birth was significantly higher than those of three other genotype groups (Fig. 4 A, K51R-Tg/Nkx2.5^+/−^ 64.71% vs. Wt 5.88%, or Nkx2.5^+/−^ 7.69% or K51R-Tg 5.56%, respectively, p<0.001), indicative of severe cardiac dysfunctions. Global heart morphology showed enlarged hearts from K51R-Tg/Nkx2.5^+/−^ mice (Fig. 4, compare E with B, C and D), with both atria glutted with blood, indicating heart failure. The subsequent histological analysis revealed the presence of compound ASD/VSD in the K51R-Tg/Nkx2.5^+/−^ mouse hearts (compare E′ with B′, C′ and D′) in 10 out of 11 demised K51R-Tg/Nkx2.5^+/−^ mice examined, and a single ASD in 1 mouse. Dead pups from other genotype groups showed either normal structure (Nkx2.5^+/−^ and K51R-Tg, respectively) or a single ASD (wt) (Fig. 4F). Thus, expression of the transgene K51R in the existence of Nkx2.5 haploinsufficiency caused CHDs, which was likely the primary cause of premature death observed in the compound K51R-Tg/Nkx2.5^+/−^ mice.

### Decreased cardiomyocyte proliferation in the compound K51R-Tg/Nkx2.5^+/−^ mouse hearts

**Figure 5:**
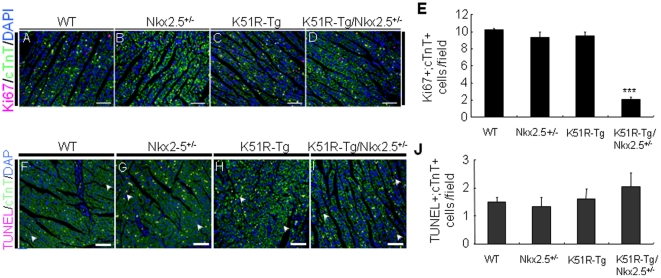
Decreased cardiomyocyte proliferation but not increased apoptosis in the compound K51R-Tg/Nkx2.5^+/−^ mouse hearts. Ki67 (A–D) and TUNEL (F–I) staining was performed on 5 µm sections of mouse hearts from four genotype groups at P7 as indicated. cTnT was indicated for cardiomyocytes, and DAPI for nucleus. Statistical analyses of assays of cardiomyocyte proliferation and apoptosis were shown in E and J, respectively. The number of double positive (Ki67+/cTnT+ or TUNEL/cTnT+) cells was scored from at least three randomly selected fields in left ventricle per animal. Arrows in F–I indicated double positive (TUNEL/cTnT+) cardiomyocytes. N = 3 for each group. ***, p<0.001. Scale bar, 100 µm.

Cardiac structural phenotypes such as ASDs and VSDs can be associated with decreased proliferation and/or increased apoptosis of cardiomyocytes [33], [34], [35]. To explore the molecular basis underlying the CHDs present in the compound K51R-Tg/Nkx2.5^+/−^ mice, cardiomyocyte proliferation in the heart sections from age-matched wt, K51R-Tg, Nkx2.5^+/−^ and compound K51R-Tg/Nkx2.5^+/−^ mice (n = 3 for each group) at P7 was first assessed using antibodies against Ki67, a cell proliferating maker [36], and cardiac troponin-T (cTnT), a cardiomyocyte marker [37]. As shown in Fig. 5 A–D, the number of proliferating cardiomyocytes (double Ki67+/cTnT+) in both left ventricle and atrioventricular septum of the compound K51R-Tg/Nkx2.5^+/−^ mice was substantially lower than those in other three genotype animals (number/field: K51R-Tg/Nkx2.5^+/−^ 2±0.67 vs. wt 10.2±1.03, Nkx2.5^+/−^ 9.3±0.95, and K51R-Tg 9.5±0.85, p<0.0001, and data not shown). Next, the same tissue sections mentioned above were stained with TUNEL reagent. The rates of double TUNEL+/cTnT+ positive cardiomyocytes were similar between these four genotypes (Fig. 5 F–G, 1–2 cells/field, p>0.05). Collectively, these findings suggested that defective cardiomyocyte proliferation but not altered apoptosis underpinned CHDs present in the compound K51R-Tg/Nkx2.5^+/−^ mice.

### Two out of four surviving compound K51R-Tg/Nkx2.5^+/−^ mice examined exhibited dilated cardiomyopathy

**Figure 6:**
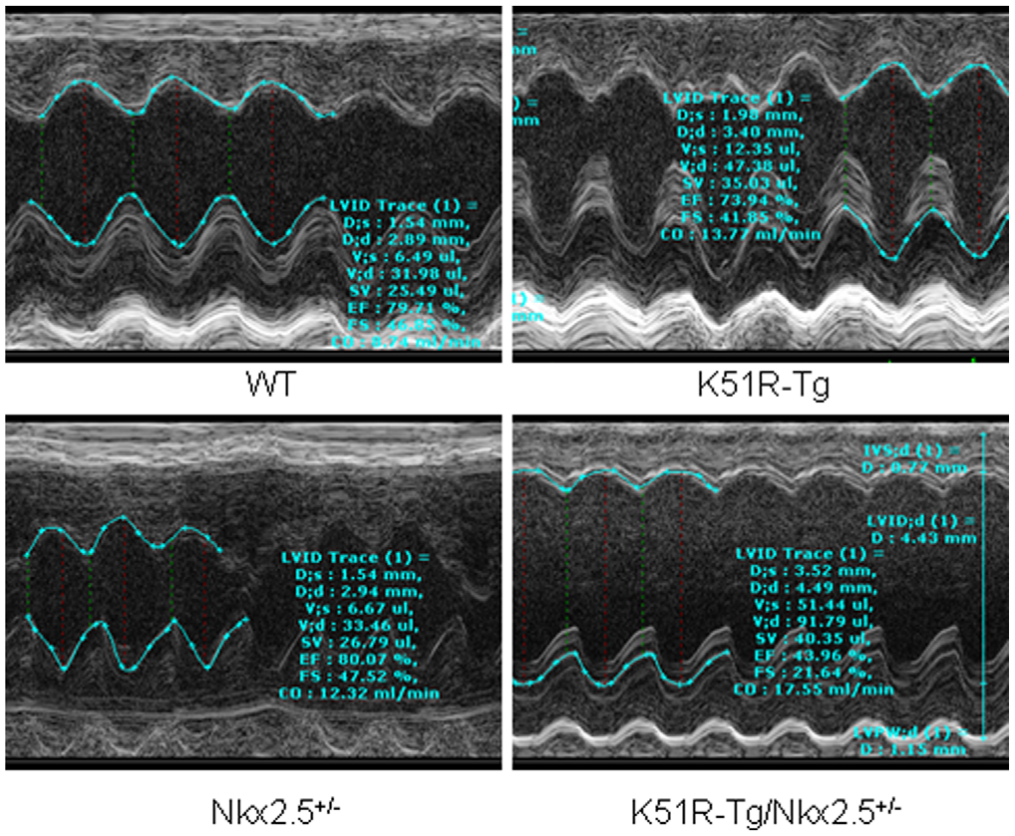
Two out of four surviving compound K51R-Tg/Nkx2.5^+/−^ mice exhibited dilated cardiac chamber and cardiac dysfunction. Representative echocardiography images from age-matched four genotype animals were shown. Note the dilated left ventricular chamber in K51R-Tg/Nkx2.5^+/−^ mouse. Red broken line: left ventricular internal diameter (LVID, diastolic); green broken line: left ventricular internal diameter (LVID, systolic). EF, ejection fraction; FS, fractional shortening.

Although ∼65% of the compound K51R-Tg/Nkx2.5^+/−^ mice died prematurely, ∼35% of them survived to adulthood. We then asked whether these mice exhibited cardiac dysfunctions. Non-invasive cardiac function analysis was carried out on surviving progenies at approximately 4 months age obtained from the cross between K51R-Tg and Nkx2.5^+/−^ mice. As shown in Table 1, the compound K51R-Tg/Nkx2.5^+/−^ mice exhibited an increase in LV volume during systole (33.08±8.42 µl vs. mice of wt 15.50±9.08 µl, Nkx2.5^+/−^ 13.78±5.67 µl, and K51R-Tg 12.32±0.83 µl, respectively), and during diastole (64.95±10.93 µl vs. mice of wt 47.75±12.68 µl, Nkx2.5^+/−^ 34.50±7.25 µl, and K51R-Tg 48.47±1.38 µl, respectively). Also, compound K51R-Tg and Nkx2.5^+/−^ mice showed a decrease in cardiac contraction capacity as indicated by both percent ejection fraction (K51R-Tg/Nkx2.5^+/−^ 51.68±7.53 vs. wt 72.32±9.33, Nkx2.5^+/−^ 65.97±9.59, and K51R-Tg 74.45±2.3, respectively) and percent fractional shortening K51R-Tg/Nkx2.5^+/−^ 26.59±4.96 vs. wt 41.75±7.26, Nkx2.5^+/−^ 36.84±7.13, and K51R-Tg 42.45±2.14, respectively). However, the difference of the above-mentioned indexes between these groups was not statistically significant due to variations among individual K51R-Tg/Nkx2.5^+/−^ mice examined. Further elaborated interrogation revealed enlarged left ventricular chamber with impaired cardiac functions in two of four compound K51R-Tg/Nkx2.5^+/−^ mice examined (Fig. 6), suggesting dilated cardiomyopathy, although the size of the sample needs to be expanded to reach a statistical significance.

**Table 1:** Cardiac functional analysis in the progenies obtained from cross-breeding between K51R-Tg and Nkx2.5^+/−^ mouse lines.

Genotypes				
	wt	Nkx2.5^+/−^	K51R-Tg	K51R-Tg/Nkx2.5^+/−^
	(n = 3)	(n = 4)	(n = 3)	(n = 4)
Diameters; s (mm)	2.01±0.47	1.91±0.37	1.97±0.05	2.85±0.35
Diameters; d (mm)	3.36±0.36	2.94±0.28	3.43±0.04	3.84±0.28
Volume; s (µl)	15.50±9.08	13.78±5.67	12.32±0.83	33.08±8.42
Volume; d (µl)	47.75±12.68	34.50±7.25	48.47±1.38	64.95±10.93
stroke volume (µl)	32.25±3.95	21.22±3.01	36.14±2.17	31.88±3.84
%EF	72.32±9.33	65.97±9.59	74.45±2.37	51.68±7.53
%FS	41.75±7.26	36.84±7.13	42.45±2.14	26.59±4.96
cardiac output (ml/min)	12.87±2.24	9.39±1.39	14.41±0.41	13.42±1.83
LV mass (mg)	123.17±12.72	111.88±18.32	113.53±11.84	128.80±16.49
BW (g)	25.25±2.80	25.25±2.33	25.73±1.95	24.65±2.95
LV mass/BW	4.60±0.42	4.60±0.93	4.40±0.16	5.30±0.51

Echocardiography was performed on animals of 4 months age as described in Materials and Methods. Note that although there was no statistically significant difference reached in all parameters shown between compound K51R-Tg/Nkx2.5^+/−^ group and the other three groups due to small size of the sample examined, there was a trend of increase in diastolic and systolic volumes, and a trend of decrease in cardiac function (%EF and %FS) in the compound K51R-Tg/Nkx2.5^+/−^ mice compared with the other group animals. EF, ejection fraction; FS, fractional shortening.

## Discussion

Nkx2.5 is a key transcription factor for embryonic cardiogenesis and its naturally occurring mutations are causally linked to human cardiac anomalies. Based on our previous findings that Nkx2.5 was a sumoylation substrate [18], in the present study we investigated the effects of CHDs-associated Nkx2.5 missense mutations on Nkx2.5 sumoylation and the impact of accumulated unsumoylatable Nkx2.5 mutant K51R on murine heart development and function.

### CHDs-associated Nkx2.5 mutants and sumoylation activity

The present study revealed that all four Nkx2.5 HD mutants and one C-terminal domain mutant C309L tested rendered either substantial or intermediate level of decrease in SUMO conjugation, respectively, while the intact principle SUMO site was existent, indicating the involvement of these sites in the regulation of Nkx2.5 sumoylation. The similar phenomena of modulation of sumoylation by non-sumoylation site(s) have been observed in other SUMO targets [38]. The diminished capability of these Nkx2.5 mutants to be sumoylated was also reflected in the target promoter activity assays, in which the presence of SUMO-1 failed to fully potentiate the activity of any of those sumoylation-deficient mutants compared with that of wild type Nkx2.5. On the other hand, the R25C mutant showed no significant changes in SUMO modification. However, it is noteworthy that the mutant R25C exhibited higher sensitivity to SUMO conjugation compared with wild type Nkx2.5, because the lower expression of SUMO-1 protein induced its sumoylation to the level equivalent to that of wild type Nkx2.5 with higher expression of SUMO protein. This increased sensitivity of R25C to sumoylation probably accounted for the higher activation of target gene promoter by R25C, possibly due to active endogenous sumoylation machinery in Hela cells. And, if this is the case, then the addition of exogenous SUMO-1 would do little to further increase its activity. However, this hypothesis needs further investigation. The higher transcriptional activity of R25C mutant observed in the present study was not observed in a previous report [5]. This discrepancy was likely attributable to the different systems in which the experiments were conducted. Increased sumoylation sensitivity was also observed in other SUMO substrates once a mutation occurred. For instance, DJ-1, a protein involved in the onset of Parkinson's disease (PD), was sumoylated on lysine residue 130 (K130) [39]. The DJ-1 mutant L166P, which was identified in human PD patients, promoted sumoylation by forming poly- or multi-SUMO-1, and/or by forming abnormal aggregates [39]. However, the altered sumoylation of L166P decreased the activity of DJ-1.

How these mutations affected Nkx2.5 sumoylation in the presence of entire principle SUMO site remains unclear. It appears that the relative distance from a mutation site to the SUMO site had no any predictable impact on Nkx2.5 sumoylation. For instance, four HD mutations and V309L, the former closer to K51 than C265Y, and the latter farther away from K51 than C265, all showed repressive effects on Nkx2.5 sumoylation. R25C, on the other hand, which is the most adjacent to K51, increased the sensitivity of Nkx2.5 to sumoylation. Possibly, these mutations caused changes of three dimensional structure of Nkx2.5, which mask the SUMO site and/or make it less accessible to SUMO pathway components (HD and C309L mutations), or expose the SUMO site better for sumoylation (R25C mutation). Alternatively, these sites are required for a direct physical contact of Nkx2.5 with SUMO pathway factors, and mutations either disrupt or enhance this contact. The above findings indicate that all HD mutations of Nkx2.5, which negatively affect its DNA binding activity, also severely affect its sumoylation. These observations point to the likelihood that various cardiac phenotypes associated with different Nkx2.5 mutants are in part attributable to the different degrees of altered sumoylation activity of Nkx2.5 mutants.

### Sumoylation-deficient Nkx2.5 K51R mutant and CHDs

Previous studies demonstrated that expressing wild type Nkx2.5 or one HD mutant I183P caused embryonic lethality or cardiac anomalies [7], [8]. In contrast to the above observations, cardiac specific expression of K51R mutant in the wild type mice was not sufficient to generate significant cardiac defects. Although K51R and those HD mutants shared certain properties such as attenuated transcriptional activity and reduced sumoylation [18], it is possible that K51R and HD mutants could behave differently in vivo. For example, K51R and HD mutants may disrupt physical association with different co-factor(s). Alternatively, it may also involve variable expression levels of the transgenes. However, in striking contrast to the lack of overt cardiac malformation in single K51R-Tg mice, a large portion of Nkx2.5 haploinsufficient mice with overexpressed K51R mutant in heart developed lethal congenital heart defects – ASD/VSD. It should be pointed out that the ASD/VSD observed in the compound K51R-Tg/Nkx2.5^+/−^ mice might not be attributable only to the defective sumoylation of K51R; the certain level of dominant negative activity of K51R mutant should also be taken into consideration [18]. However, the current findings support the notion that K51R mutant could mimic those sumoylation deficient CHDs-associated Nkx2.5 mutants in vivo to generate cardiac structural defects in the condition of Nkx2.5 haploinsufficiency.

The exact mechanism(s) by which K51R mutant caused CHDs in the presence of one wild type Nkx2.5 allele remains unclear. Apparently, apoptosis was not involved, despite it is a critical process for cardiac development and function. Although normal cardiomyocyte proliferation was observed in the hearts of Nkx.2.5 null embryos at embryonic day (E) 10.5 [1], overexpression of wild type Nkx2.5 did increase cardiomyocyte division [40]. Given the finding that K51R mutant inhibited the transcriptional activity of wild type Nkx2.5 partially via competing with Nkx2.5 dimerization [18], the observation of substantially suppressed cardiomyocyte proliferation in the compound K51R-Tg/Nkx2.5^+/−^ mice indicated a positive role of Nkx2.5 in cardiomyocyte division. Whether this diminished cardiomyocyte proliferation was solely responsible for the development of CHDs present in the compound K51R-Tg/Nkx2.5^+/−^ mice requires further interrogation.

One of the limitations of the approach used in the present study is that cardiac α-MHC controlled K51R expression was restricted to myocardium, therefore the anomalies involving great vessels such as double outlet right ventricle (DORV) and Tetralogy of Fallot (TOF) that were seen in human patients associated with heterozygous Nkx2.5 mutants were not expected/observed in our model. Thus, it will be interesting to see if K51R knockin in murine model would lead to the development of great vessel defects that resemble those observed in human patients.

### SUMO and cardiovascular disorders

Involvement of the SUMO pathway in a number of pathogenic processes such as the development of craniofacial defects, neurodegenerative disease progression and cancer development has been considerably investigated [41], [42], [43], however, evidence for the role of SUMO in the development of cardiovascular disorders is newly emerging. SUMO targets a number of transcription factors, such as serum response factor (SRF) [44], GATA4 [45], [46], and myocardin [25], which are essential for normal cardiac morphogenesis and function [20]. It is important to note that the covalent attachment of SUMO to the above-mentioned factors positively regulated their functions, counter to the general belief that sumoylation serves as a negative regulator of the activity of substrates. Recently, two naturally occurring sumoylation-defective mutants of the nuclear structural protein, lamin A, , were proposed to be potentially linked to human familial dilated cardiomyopathy [47]. Similarly, the present study demonstrated altered sumoylation status in a number of human Nkx2.5 mutants that are implicated in CHDs. Moreover, expression of sumoylation deficient Nkx2.5 mutant in the mice with one wild type Nkx2.5 allele caused CHDs. Given the findings of CHDs in the SUMO-1 mutant mice [22], and identification of a unique mutation in the *cis*-regulatory sequence of the SUMO-1 gene in a patient with both cleft lip/palate and ASD [22], our studies further indicate a significant influence of the SUMO pathway in the cardiovascular diseases. With the advances in the phenotypic analysis of additional animal models with genetic modifications that specifically target the SUMO conjugation machinery, we believe that the essentiality of an intact SUMO conjugation pathway for normal cardiac structural morphogenesis and function will be clarified.
